# LDexpress: an online tool for integrating population-specific linkage disequilibrium patterns with tissue-specific expression data

**DOI:** 10.1186/s12859-021-04531-8

**Published:** 2021-12-20

**Authors:** Shu-Hong Lin, Rohit Thakur, Mitchell J. Machiela

**Affiliations:** 1 Integrative Tumor Epidemiology Branch, Division of Cancer Epidemiology and Genetics, National Cancer Institute, Rockville, MD 20892 USA; 2 Laboratory of Translational Genomics, Division of Cancer Epidemiology and Genetics, National Cancer Institute, Rockville, MD 20892 USA

**Keywords:** Linkage disequilibrium, Gene expression, Genome-wide association study, LDexpress, LDlink, Quantitative trait locus

## Abstract

Genome-wide association studies have identified thousands of genetic susceptibility loci associated with cancer as well as other traits and diseases. Mapping germline variation in identified genetic susceptibility regions to alterations in nearby gene expression nominates candidate genes potentially related to disease risk for further functional investigation. We developed LDexpress as an online resource that integrates population-specific linkage disequilibrium data from the 1000 Genomes (1000G) project and tissue-specific expression data from the Genotype-Tissue Expression project to better study regional germline variation impacting gene expression. LDexpress is a publicly available web tool designed to be easy to use, flexible to conduct a wide range of variant queries, and quick to efficiently investigate dozens of query variants across multiple tissue types. We demonstrate the utility of LDexpress using example genomic queries and anticipate this tool will accelerate understanding of disease etiology by uncovering associations of regional germline variation to nearby gene expression.

## Background

Genome-wide association studies (GWAS) have made tremendous advances in identifying germline genetic variation associated with disease risk [1]. Identifying a susceptibility locus associated with disease risk, however, is only the first of many steps needed for understanding the etiologic mechanisms by which germline variation impacts a quantitative trait or confers altered disease risk. Regional fine mapping studies are needed for surveying surrounding germline variation, disentangling linkage disequilibrium (LD) patterns, and nominating nearby candidate genes with potential functional relevance.

A common approach for selecting nearby candidate genes for functional investigation is by performing an expression quantitative trait locus (eQTL) analysis. eQTL analyses identify germline variants associated with local gene expression by testing for a relationship between variant genotype and gene expression levels [2–4]. While many gene expression datasets have been created, the Genotype-Tissue Expression (GTEx) project [5] is the most comprehensive resource to date with expression data on 54 non-diseased tissue sites collected from nearly 1000 individuals. In addition to gene expression data, the GTEx project has publicly available dense genotype data on over 4.6 million variants for conducting eQTL analyses.

GTEx is an excellent resource for eQTL information but lacks information on local linkage disequilibrium patterns. Linkage disequilibrium (LD) is the nonrandom distribution of alleles at nearby loci in a defined population [6]. Germline variants that are in high LD will have correlations between alleles that are often inherited together on the same underlying haplotype. As the pattern of LD varies by ancestral population, eQTL relationships will likewise vary by ancestral population. Whole-genome sequencing data from the 1000 Genomes (1000G) Project [7] on 2504 individuals across 26 populations allows for easy estimation of population-specific LD patterns as implemented in LDlink [8]. LDexpress (https://ldlink.nci.nih.gov/?tab=ldexpress) is a new open access web-based tool that seamlessly integrates LD patterns estimated from the 1000G project with gene expression data from GTEx v8 to investigate correlations between genotype and regional gene expression.

### Construction and content

LDexpress is designed to be accessible to a wide range of genomic investigators interested in disengangling tissue- and population-specific eQTL associations. The landing page for accessing LDexpress (https://ldlink.nci.nih.gov/?tab=ldexpress) asks the user to input information for the desired query including a list of RefSeq (RS) numbers or genomic positions of query variants, the 1000G population of interest, the GTEx tissue or tissues of interest, threshold options for LD metrics (e.g., R^2^ or D′), GTEx eQTL p-value, and genomic window of interest. The calculate button submits the form data to the LDexpress Python flask server which pulls phased VCF data (Phase 3, Version 5) for the 1000G population of interest and calculates LD measures for sequenced variants in the selected window surrounding the query variants. LD measures are calculated as previously described in LDlink [8]. Variants that surpass the user defined LD threshold are kept and subsequently queried in a local MongoDB GTEx database (https://storage.googleapis.com/gtex_analysis_v8/single_tissue_qtl_data/GTEx_Analysis_v8_eQTL.tar, accessed 8/19/2020) indexed by variant position for rapid retrieval. eQTL associations that match the user defined GTEx tissue of interest and meet the user provided p-value significance value are returned to the LDexpress web interface. Most variant queries take less than 10 seconds to complete.

LDexpress organizes query results into a menu of clickable links for each query variant that has an eQTL association in LD within a selected GTEx tissue. The variant links will direct the user to a searchable and sortable table that provides details on variants in LD that have eQTL associations in GTEx. The table displays information on variant RS number, position and LD measures with the query variant as well as provides details on the eQTL association including gene symbol, Gencode ID, tissue type, effect size and p-value. Custom links are also generated to dbSNP, NCBI Gene, Ensembl and GTEx where further details and plots on the variants, genes and eQTL association are provided. Variants with no eQTL associations in LD in GTEx will be displayed as warnings. All LDexpress returned results can also be downloaded in a tab-delimited text file for further investigation. LDexpress web content is programmed in HTML5 for cross platform compatibility. The LDexpress web tool is available and fully supported for two full years following publication and does not require mandatory registration to use the resource. LDexpress can also be accessed through the LDlink API (https://ldlink.nci.nih.gov/?tab=apiaccess) using the options described above to allow batch queries.

### Utility and discussion

**Fig. 1:**
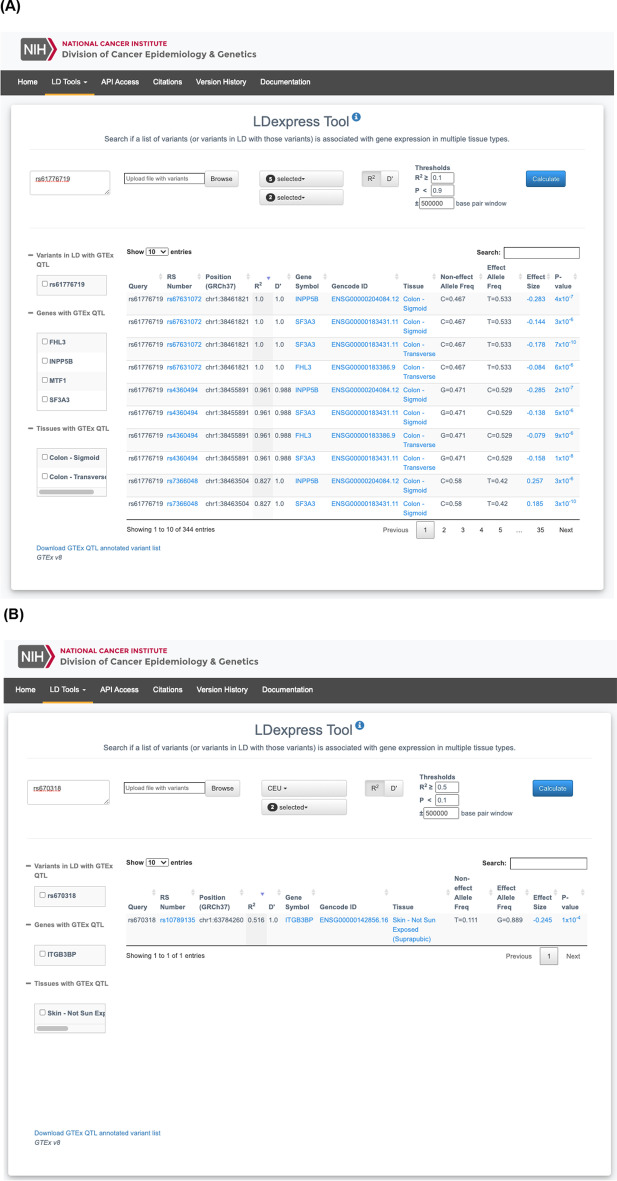
Screenshots of results from example LDexpress queries. Two genome-wide significant loci from recent colorectal cancer (**A**) and cutaneous melanoma (**B**) GWAS were selected as input (rs61776719 and rs670318, respectively)

As an example of LDexpress use cases, a few sample queries are provided. The first is from a GWAS of colorectal cancer risk that identified a susceptibility locus at 1p34.3 tagged by rs61776719 [9]. The original publication did not identify eQTLs using samples from the INTERMPHEN study, and the tagging variant rs61776719 was not found in GTEx. LDexpress was used to query rs61776719 and identify potential genes with altered expression in colon tissue (Fig. 1A). The resulting query using all 1000 Genomes European (EUR) populations identified two variants in high LD (R^2^ > 0.9; rs4360494 and rs67631072) that were associated with altered expression of *SF3A3*, *INPP5B*, and *FHL3* in transverse and sigmoid colon tissues. The second example query is from a recent cutaneous melanoma GWAS [10] which identified a locus at 1p31.3 tagged by rs670318. A simple GTEx query identifies no skin tissue eQTLs for rs670318. However, LDexpress using the Utah Residents with Northern and Western European ancestry (CEU) population as a reference identifies additional GTEx variants in LD with rs670318 which have eQTL associations with the *ITGB3BP* gene in skin tissue (Fig. 1B). A similar LDexpress query for rs670318 in the Yoruba in Ibadan, Nigera (YRI) population does not identify additional LD surrogates in GTEx, highlighting the impact of the selected ancestry reference panel for identifying LD surrogates.

LDexpress is a user-friendly tool to facilitate the identification of candidate genes regulated by disease-associated germline susceptibility loci. LDexpress provides a novel approach for merging population-specific LD data with tissue-specific gene expression data to identify local associations between germline variation and gene expression with relevance for disease etiology.

We showcase the utility of LDexpress by identifying eQTLs with *SF3A3*, *INPP5B*, and *FHL3* at the 1p34.3 colorectal cancer risk locus tagged by rs61776719. *SF3A3* is a subunit for splicing factor 3a which interacts with cellular stress response 1 (*CSR1*), a tumor suppressor gene. *CSR1* binding leads to migration of *SF3A3* to cytoplasm and reduces splicing efficiency of epidermal growth factor receptor and platelet-derived growth factor receptor [11]. The Human Protein Atlas (http://www.proteinatlas.org) also suggested that the expression of *SF3A3* protein might be a prognostic factor in liver cancer [12]. *INPP5B* is a member of inositol polyphosphate-5-phosphatase family which regulates calcium signaling. The expression of *INPP5B* protein is prognostic for renal, head and neck, and pancreatic cancers in The Human Protein Atlas. Finally, *FHL3* has been shown to modulate *SOX4* expression leading to abrogation of self-renewal and epithelial-mesenchymal transition in cancer cells [13, 14].

In the melanoma example, LDexpress demonstrated ancestry and tissue-specific identification of eQTLs by finding variants in linkage disequilibrium at 1p31.3 with the tagging rs670318 variant. The ancestry-specific population groups in the 1000 Genome Project sequencing data used by LDexpress enables cancer researchers to further disentangle potential differences in cancer risk based on genetic ancestry. The identification of an eQTL with integrin subunit beta 3 binding protein (*ITGB3BP*) suggest this transcriptional coregulator that binds to and enhances the activity of members of the nuclear receptor families could be important for melanoma risk at 1p31.3. Functional studies in breast cancer cells indicates *ITGB3BP* induces apoptosis through a caspase 2-mediated signaling pathway.

In the current LDexpress implementation, users are allowed to query LD patterns for specific ancestral groups. However, eQTLs were derived mostly from indviduals of European decent. Out of 838 GTEx participants, 103 were African American, 12 were Asian American, and the remainder (N = 723) were White. Previously, population-biased eQTL (pb-eQTL) were examined in GTEx across 31 tissues and discovered only 178 pb-eQTL suggesting that ancestry likely accounts for a limited proportion of all eQTLs [15]. Despite the efforts to account for ancestry in GTEx by adjusting for genotyping principal components in statistical modeling, the sample size of GTEx is limited in the ability to identify eQTL specific for non-European groups. We will closely monitor updates from GTEx and other eQTL databases and implement further options in LDexpress for pb-eQTL should public resources become available.

## Conclusions

Our example LDexpress queries showcase the convenience and potential etiologic insights LDexpress can add to post-GWAS analyses that is not easily accomplished with simple GTEx queries, which are unable to perform comprehensive population-specific searches for proxy variants in LD. We anticipate LDexpress to be a useful tool for researchers that assists with identifying potential genes relevant for disease risk for future functional investigation.

## Data Availability

The datasets supporting the conclusions of this article are available in https://www.gtexportal.org and https://www.internationalgenome.org/. The data used for the analyses described in this manuscript were obtained from the GTEx Portal on 19/08/2021 and dbGaP accession number phs00424.v5.p1 on 19/08/2021.

## Abbreviations

GWAS: Genome-wide association studies
1000G: 1000 Genomes
GTEx: The Genotype-Tissue Expression
LD: Linkage disequilibrium
eQTL: Expression quantitative trait locus
RS: RefSeq
EUR: European
CEU: Utah Residents with Northern and Western European ancestry
YRI: Yoruba in Ibadan, Nigera
ITGB3BP: Integrin subunit beta 3 binding protein
CSR1: Cellular stress response 1
